# The Role of Sex in Individual and Group Rowing Performance

**DOI:** 10.3390/sports14040161

**Published:** 2026-04-17

**Authors:** Juan Gavala-González, Juan Gamboa González, José Carlos Fernández-García, Elena Porras-García

**Affiliations:** 1 Researching in Sport Science: Research Group (CTS-563) of the Andalusian Research Plan, University of Malaga, 41003 Malaga, Spain; jgavala@us.es (J.G.-G.); jcfg@uma.es (J.C.F.-G.); meporgar@upo.es (E.P.-G.); 2 Department of Physical Education and Sports, University of Seville, 41013 Seville, Spain; 3 Department of Physical Education and Sport, Cardenal Spínola, CEU San Pablo Andalucía University, 41930 Sevilla, Spain; 4 Department of Didactics of Languages, Arts and Sport, University of Malaga, Andalucía-Tech, Instituto de Investigación Biomédica de Málaga (IBIMA), 29071 Malaga, Spain; 5 Department of Physiology, Anatomy and Cellular Biology, University of Pablo de Olavide, 41013 Seville, Spain

**Keywords:** rowing performance, sex differences, Ringelmann effect, Köhler effect, Borg scale

## Abstract

This study analysed the potential influence of crew size on performance (stroke rate, strokes/min; distance travelled, m/min; and average power, W), physiological responses (post-exercise heart rate and heart rate measured three minutes after exercise) and perceptual responses (Borg scale). A total of 136 adolescent athletes (100 males and 36 females; mean age = 15.79 ± 1.14 years) performed four three-minute maximal-effort trials on a rowing ergometer across four conditions: individual trials (C1), two-person crews (C2), four-person crews (C3), and eight-person crews (C4). Results showed a significant increase in stroke rate (strokes/min) in both sexes as crew size increased (C1 33.16 ± 2.54 vs. C4 34.19 ± 2.21 strokes/min; C1–C4 *p* = 0.01; C2–C4 *p* = 0.003). Men reported greater perceived exertion in C1 compared with C4 (Borg 7.80 ± 0.79 vs. 7.46 ± 0.74; *p* = 0.032), despite no associated changes in performance (863.88 ± 45.10 vs. 863.26 ± 47.63 m/min) or average power (311.71 ± 46.43 vs. 311.44 ± 50.43 W), whereas no differences in perceived exertion were observed in women (Borg 7.59 ± 0.84 vs. 7.56 ± 0.76). Cardiovascular responses were similar across sexes and experimental conditions. In summary, these preliminary findings could point toward the existence of sex-differentiated patterns. The data appear to suggest a more pronounced tendency toward the ‘crew-size effect’ among the men in the sample, whereas an inclination toward maintaining individual responsibility is observed in the women.

## 1. Introduction

Sport performance results from the interaction of multiple factors, predominantly physiological, psychological and social, whose contribution varies according to sex, training level, and task execution. In endurance sports, average sex differences have been described in variables associated with performance (e.g., muscle mass, haemoglobin concentration and maximum oxygen consumption) [1,2,3,4,5], although their expression depends on the task, the duration of effort, and the degree of training [6,7]. Within this framework, the subjective perception of exertion (RPE) is a relevant integrative indicator [8,9] because it reflects the interaction between physiological signals and cognitive–emotional processes and contributes to regulating motor behaviour during exercise [10]. Beyond individual factors, group contexts can also modify effort and performance.

The Ringelmann effect refers to the decline in individual contribution as group size increases, a pattern commonly attributed to coordination losses and social loafing [11,12]. In contrast, the Köhler effect suggests that, under conditions of functional interdependence and a perception of indispensability, individuals may increase their effort when working in groups, especially those individuals with lower relative performance [13,14].

In experimental designs applied to physical performance, behavioural patterns compatible with the Ringelmann effect would be expressed as reductions in individual performance (e.g., power or distance) as group size increases, whereas Köhler-compatible patterns would involve maintained or increased effort in group conditions (e.g., power/distance and/or RPE), particularly among participants with lower relative performance.

Rowing may represent a suitable activity for examining these dynamics, as it combines high physiological demands with requirements for synchronisation and cooperation within the crew. Although rowing research has traditionally focused on biomechanical and physiological aspects, psychosocial variables, such as cohesion and collective efficacy, have also been shown to be relevant for performance in cooperative tasks [15,16,17]. In training categories, it has also been observed that individual performance and team performance may differ according to the context, reinforcing the need to analyse both performance levels in an integrated manner [18]. From an applied perspective, understanding how maximal performance differs between individual and collective conditions could inform the design of ergometer training sessions and the interpretation of individual performance in crew contexts.

However, empirical evidence jointly examining sex and group size in maximal-effort contexts remains limited [19], particularly in young rowers. The use of the ergometer enables task standardisation and the recording of objective variables (e.g., power, distance, and stroke rate) [20], together with physiological and perceptual responses, although mechanical interdependence is not identical to that observed in an on-water boat [21,22].

Accordingly, the present study aimed to investigate sex-related differences in performance, physiological responses, and perceived exertion among young rowers, during maximal-effort trials performed individually and in groups of different sizes (1, 2, 4, and 8 athletes), and to explore whether group-size effects on performance are compatible with Ringelmann- or Köhler-type patterns, depending on sex. It was hypothesised that: (i) group size would influence execution strategy, particularly stroke rate; (ii) objective performance outcomes (power output and distance covered) would show group-size-related changes that could reflect either Ringelmann-compatible decreases or Köhler-compatible maintenance/improvement; (iii) RPE would differ between individual and group conditions, reflecting changes in perceptual regulation; and (iv) the magnitude and direction of these effects would differ between female and male rowers.

## 2. Materials and Methods

### 2.1. Participants

This experimental study included 136 competitive rowers (100 males and 36 females). All participants had competed at the national championship level and presented a mean rowing experience of 4.21 ± 2.02 years, together with a mean age of 15.79 ± 1.14 years. They belonged to the two largest and most successful rowing clubs in Spain, as determined by performances at national championships, and the sample accounted for approximately 75% of all nationally competitive rowers within these age categories.

To be eligible for inclusion, participants were required to attend at least 90% of their scheduled weekly training sessions (four sessions per week). Athletes who did not satisfy this criterion were excluded from the study.

For the purposes of the study, the rowing clubs were contacted and several meetings were conducted with the athletes’ coaches in order to ensure consistency in training loads and crew selection procedures. Data were collected during the weeks leading up to the Spanish Autonomous Community Championships, a phase in which athletes compete not for their clubs, but for their regional federation as a unified team, thus facilitating standardisation across participants.

### 2.2. Ethical Approval

The study received approval from the Ethics Committee of the University of Pablo de Olavide (reference number 24/8-31) and was carried out in accordance with the ethical considerations for Sport and Exercise Science Research [23] and the principles set out in the Declaration of Helsinki [24], which establish the ethical standards governing research involving human participants.

Before the study was undertaken, both the athletes and their parents/guardians were informed about the objectives of the research and were asked to provide written informed consent for participation and for the publication of the results.

### 2.3. Materials and Instruments

All participants performed the tests on Concept2 Model D rowing ergometers (resistance factor set at 90). Each ergometer was equipped with a PM5 display, which enabled real-time data recording and storage, including total exercise duration, 500 metre split times, total distance covered, stroke rate and power.

In addition to these variables, participant sex, perceived exertion assessed using the Borg scale [9,25], and heart rate were recorded. Heart rate was measured using a Polar H9 chest band connected to a tablet running the Polar application.

### 2.4. Experimental Design

Participants were classified into two categories (men and women) and completed four three-minute trials: individual trials (C1), two-person crews (C2), four-person crews (C3), and eight-person crews (C4), reflecting the crew sizes used in conventional rowing. The three-minute duration was chosen because it corresponds to the time typically required for athletes at this level to complete a 1000 m distance, which is the official race distance for these age categories.

During the experiment, the PM5 displays of all ergometers were concealed. In the crew trials, the ergometers were positioned in a straight line, one behind another, in order to reproduce the spatial arrangement of rowers in an on-water boat.

### 2.5. Procedure

All tests were performed at the athletes’ habitual training facilities, following the established protocol, and a 48 h recovery period was allowed between trials [18].

Each testing session commenced with the athletes’ usual warm-up, followed by a detailed explanation of the testing procedures. On each testing day, the coaches highlighted the importance of these trials for crew selection in official competitions and encouraged the athletes to exert themselves maximally. For the crew trials, participants were informed that evaluation would be based exclusively on overall crew performance, defined as the combined total distance covered by all rowers.

In the crew trials (C2, C3 and C4), crews were formed with the aim of grouping rowers of comparable performance level. For this purpose, crew composition was determined according to the results achieved by each rower in the individual trial (C1).

Performance data were obtained from the PM5 monitor and included the total distance rowed during the three-minute trial, stroke rate, and mean power output (W). Physiological variables, namely heart rate recorded immediately after the trial and three minutes post-exercise, were collected using the Polar application. Perceived exertion was evaluated using the modified Borg scale.

### 2.6. Data Analysis

The aim of this study was to examine whether individual effort varied according to crew size. To address this aim, a repeated-measures ANOVA with one between-subjects factor was conducted. Measurements collected across the different test conditions (performance, heart rate, and perceived exertion) were considered within-subject factors, while sex was included as a between-subjects factor.

Ninety-five per cent Confidence intervals were determined for differences in performance-related variables, namely stroke rate (strokes/min), distance covered (m/min), and power (W). Heart rate was measured immediately after each test and again three minutes following exercise completion (beats per minute), and perceived exertion was assessed using the Borg scale. In all instances, Student–Newman–Keuls post hoc tests were applied, together with Bonferroni corrections used for pairwise comparisons. *p*-values < 0.05 were considered statistically significant. Unless otherwise indicated, data are presented as mean ± SEM (standard error of the mean).

The Munchly sphericity test was calculated to assess whether the assumption of equal variances of the differences was met. In cases where this sphericity assumption was violated (*p* < 0.05), the Greenhouse–Geisser epsilon (GG ε) was measured to determine whether the Huynh–Feldt correction (if GG ε ≥ 0.75) or the Greenhouse–Geisser correction (if GG ε < 0.75) should be used, and to adjust the degrees of freedom accordingly.

In all cases, the observed statistical power (1 − β) and the effect size (η^2^) were also measured, using Cohen’s criteria to determine whether the effect size was small (η^2^ ≈ 0.01), medium (η^2^ ≈ 0.06) or large (η^2^ ≈ 0.14).

All variables were analysed using the SPSS statistical package (version 25; IBM Corp., Armonk, NY, USA).

## 3. Results

After computing descriptive statistics, correlations were examined between performance variables, operationalised as stroke rate (strokes/min) across the four test conditions (C1, individual; C2, two-person crews; C3, four-person crews; and C4, eight-person crews), and the remaining outcomes, including end-exercise heart rate, heart rate three minutes post-exercise, and perceived exertion assessed using the Borg scale.

**Figure 1:**
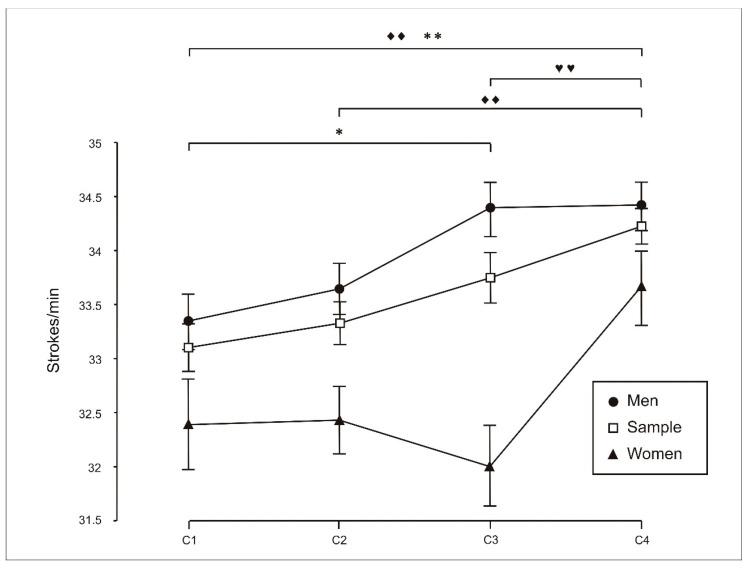
Analysis of exercise performance (stroke rate, strokes/min) during individual trials (C1), two-person crews (C2), four-person crews (C3) and eight-person crews (C4). Pairwise comparisons using the Bonferroni correction indicate the following: ♦♦, significant differences for the total sample (*p* < 0.01); *, significant differences for the men (*p* < 0.05); **, significant differences for the men (*p* < 0.01); ♥♥, significant differences for women (*p* < 0.01).

When changes in stroke rate (strokes/min) across conditions were analysed (Figure 1), significant differences were observed for the total sample (C1 vs. C4: *p* = 0.01; C2 vs. C4: *p* = 0.003), for females (C3 vs. C4: *p* = 0.004), and for males (C1 vs. C3: *p* = 0.041; C1 vs. C4: *p* = 0.01).

**Figure 2:**
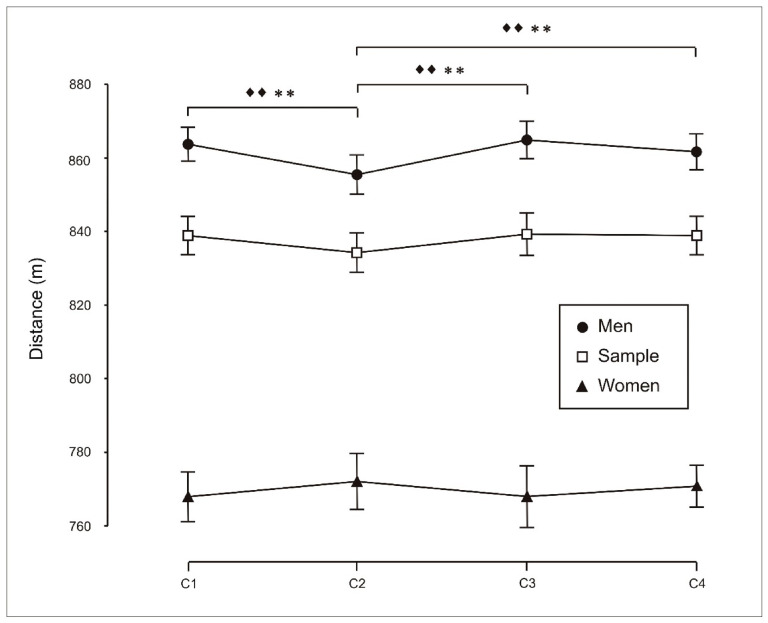
Analysis of exercise performance (distance covered, metres) during individual trials (C1), two-person crews (C2), four-person crews (C3) and eight-person crews (C4). Pairwise comparisons using the Bonferroni correction indicate the following: ♦♦, significant differences for the total sample (*p* < 0.01); **, significant differences for men (*p* < 0.01).

**Figure 3:**
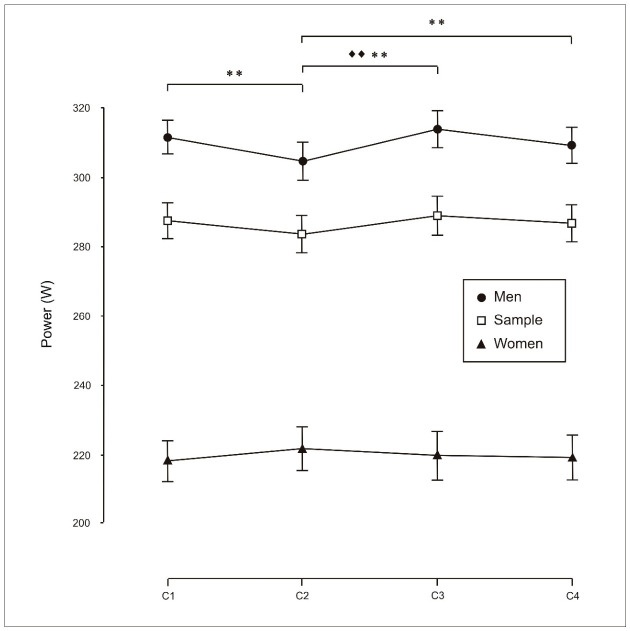
Analysis of exercise performance (mean power output, W) during individual trials (C1), two-person crews (C2), four-person crews (C3) and eight-person crews (C4). Pairwise comparisons using the Bonferroni correction indicate the following: ♦♦, significant differences for the total sample (*p* < 0.01); **, significant differences for men (*p* < 0.01).

However, no significant differences were observed among females for performance measured as distance covered (Figure 2) or mean power output (W; Figure 3), whereas significant differences were observed in males and in the total sample.

**Figure 4:**
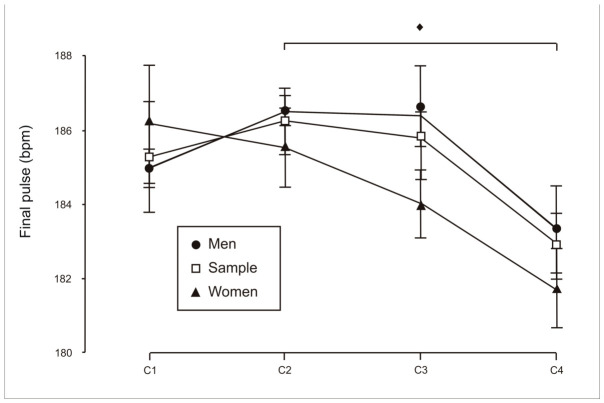
Analysis of end-exercise heart rate (bpm) during individual trials (C1), two-person crews (C2), four-person crews (C3) and eight-person crews (C4). Pairwise comparisons using the Bonferroni correction indicate significant differences for the total sample (♦, *p* < 0.05).

Regarding effort, a statistically significant effect was found in end-exercise heart rate for the total sample (Figure 4); however, these differences were no longer evident after stratifying by sex. Heart rate recorded three minutes after exercise cessation did not differ across conditions (repeated-measures ANOVA; *p* > 0.05), either in the total sample or when stratified by sex (see Table 1 and Table 2). Although the participants were relatively young and had limited sport-specific experience, they appeared to be highly aware of their exertion level and consistently performed at maximal effort across trials, as reflected by the absence of differences in end-exercise heart rate between conditions.

**Figure 5:**
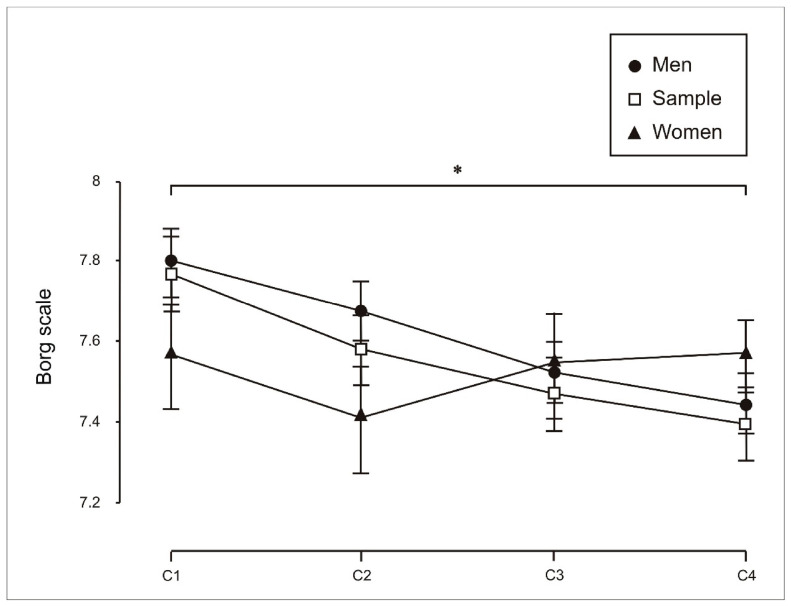
Perceived exertion (Borg scale) during individual trials (C1), two-person crews (C2), four-person crews (C3) and eight-person crews (C4). Pairwise comparisons using the Bonferroni correction indicate significant differences for men (*, *p* < 0.05).

With respect to perceived exertion (Figure 5), no significant differences were observed across conditions for either the total sample or the female subgroup. In contrast, males reported significantly higher perceived exertion in the individual condition than in the eight-person crew condition (C1 vs. C4: *p* = 0.032).

To further examine the preceding results, a repeated-measures ANOVA with condition (C1–C4) as the within-subject factor was conducted, and Mauchly’s test of sphericity indicated a violation of the sphericity assumption (*p* < 0.05; Table 2). Because Greenhouse–Geisser epsilon exceeded 0.75 in all cases, the Huynh–Feldt correction was employed. Significant effects of condition emerged for the performance measures (stroke rate, metres per minute, and power), end-exercise heart rate (bpm), and Borg RPE (all *p* < 0.01, except Borg RPE, where *p* < 0.05). In contrast, heart rate recorded three minutes post-exercise was not significantly affected (*p* = 0.232). Based on Cohen’s criteria, large effect sizes (η^2^) were observed only for end-exercise heart rate (η^2^ = 0.18). With the exception of heart rate measured three minutes post-exercise and perceived exertion, all analyses produced high statistical power (1 − β), suggesting a high probability of identifying true effects.

Significant sex differences were observed for performance expressed as metres per minute (*p* < 0.01; Table 2, Figure 2), although the associated effect size was small (η^2^ = 0.034). To complement these analyses, correlations were computed between study variables that showed meaningful associations.

**Figure 6:**
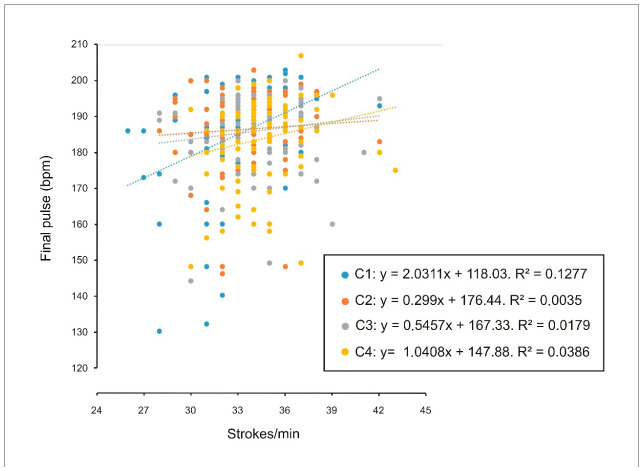
Relationship between end-exercise heart rate (beats per minute) and performance (stroke rate, strokes/min) in male participants across individual trials (C1), two-person crews (C2), four-person crews (C3), and eight-person crews (C4). A weak correlation was identified in C1 (R^2^ = 0.13). bpm = beats per minute.

**Figure 7:**
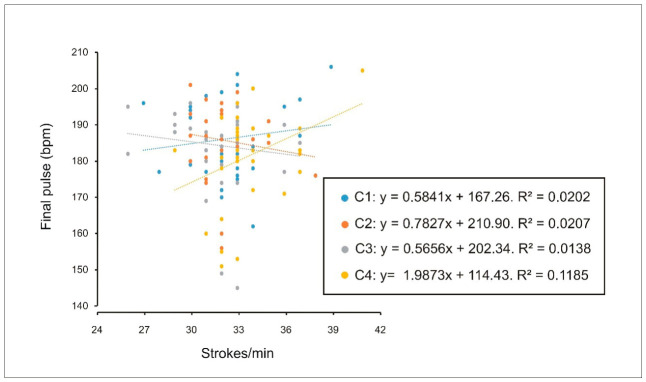
Correlation between end-exercise heart rate (beats per minute) and performance (stroke rate, strokes/minute) in women during individual trials (C1), two-person crews (C2), four-person crews (C3) and eight-person crews (C4). A weak correlation was observed in C4 (R^2^ = 0.12). bpm = beats per minute.

A weak correlation was observed between stroke rate and end-exercise heart rate in the individual condition (C1) for males (Figure 6) and in the eight-person crew condition (C4) for females (Figure 7). This pattern suggests that males tend to increase stroke rate to a greater extent in the individual condition, whereas females do so more in the crew condition. Accordingly, these findings may be interpreted as consistent with a Ringelmann-type pattern in males and a Köhler-type pattern in females.

**Figure 8:**
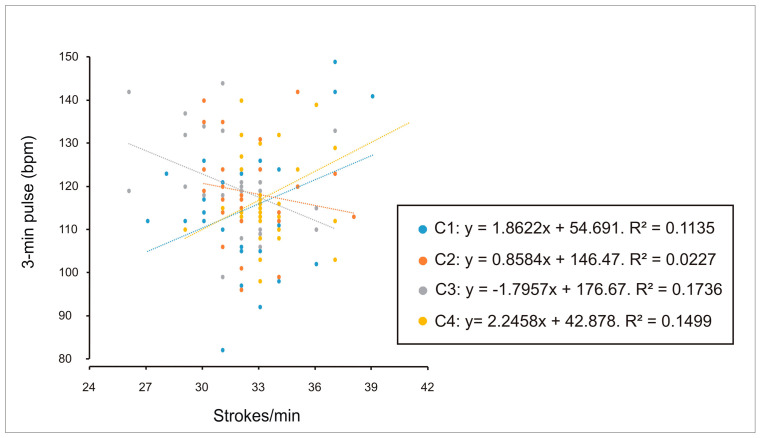
Correlation between heart rate measured three minutes post-exercise (beats per minute) and performance (stroke rate, strokes/min) in women during individual trials (C1), two-person crews (C2), four-person crews (C3) and eight-person crews (C4). Weak correlations were observed in C1 (R^2^ = 0.12), C3 (R^2^ = 0.17) and C4 (R^2^ = 0.15). bpm = beats per minute.

In females, weak correlations were observed for heart rate measured three minutes after exercise cessation in the individual condition (C1) and the crew conditions (C3 and C4; Figure 8), whereas no correlations were observed in males).

**Figure 9:**
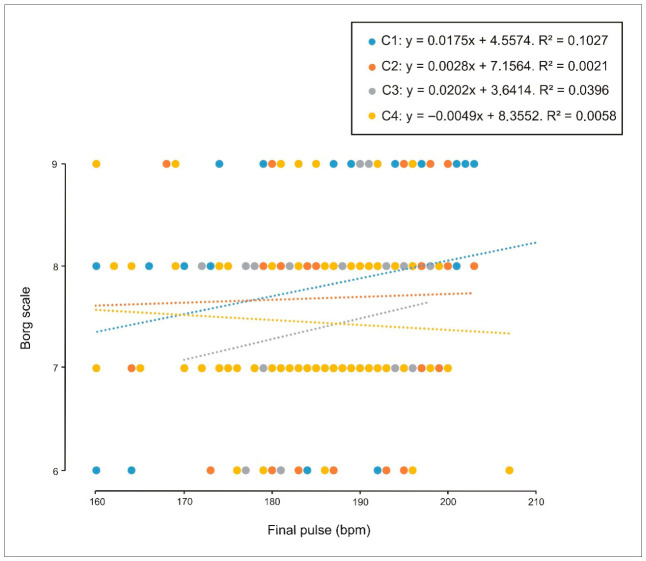
Relationship between perceived exertion (Borg scale) and performance (stroke rate, strokes/min) in male participants across individual trials (C1), two-person crews (C2), four-person crews (C3), and eight-person crews (C4). A weak correlation was identified in C1 (R^2^ = 0.10).

**Figure 10:**
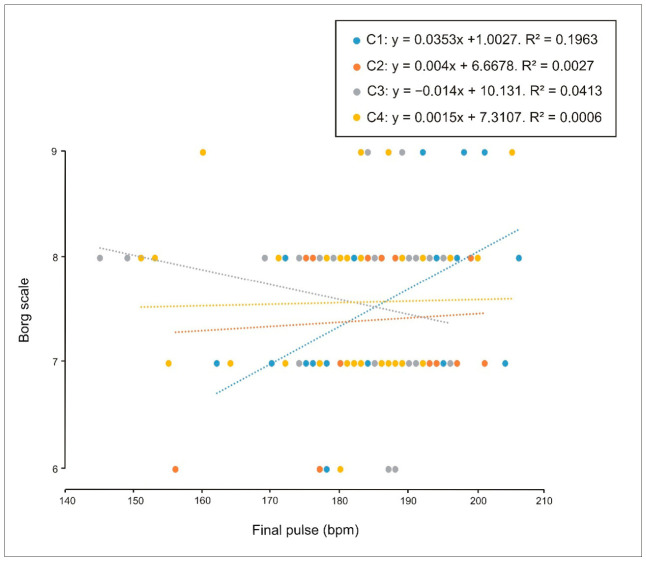
Correlation between perceived exertion (Borg scale) and performance (stroke rate, strokes/min) in women during individual trials (C1), two-person crews (C2), four-person crews (C3) and eight-person crews (C4). A weak correlation was observed in C1 (R^2^ = 0.20).

Finally, with respect to perceived exertion, weak correlations were observed in both males (Figure 9) and females (Figure 10) between Borg RPE and end-exercise heart rate in the individual condition (C1). This pattern might suggest that, as effort is shared under crew conditions (C2, C3 and C4), exertion is perceived as lower. No correlations were observed between sex, performance (stroke rate), and perceived exertion.

## 4. Discussion

This section discusses the findings in relation to group-size effects in youth rowing, while considering possible theoretical interpretations and their practical implications for training and crew management

The findings suggest that the effects of different crew size conditions on performance are not consistent across variables. Although clear sex differences were observed in absolute performance (distance and power) [26], changes between conditions were generally modest and characterised by small effect sizes. This suggests that crew configuration influences how performance is expressed, without constituting a consistent pattern of gain or loss that can be directly attributed to social effects. This profile is consistent with the multifactorial nature of performance in endurance sports and suggests that athletes may adjust their behaviour when the execution context changes [1,7,10,27].

In contrast, stroke rate was one of the variables most consistently affected by the condition. Cadence tended to increase as crew size increased, particularly in the eight-person configuration. These findings suggest that young rowers adapted both their pacing behaviour and technical execution according to the structure of the task and the size of the crew.

However, as described in the classical literature on group performance, changes in collective context may induce adjustments in coordination and strategy that do not necessarily translate into proportional mechanical gains [11,12]. In line with this interpretation, increases in stroke rate were not accompanied by systematic improvements in distance or power, suggesting that a higher cadence may reflect context-dependent adjustments in execution or coordination demands rather than an effective strategy for increasing net performance.

Overall, cardiovascular responses remained consistent across conditions, especially when heart rate was evaluated three minutes after exercise. While small differences were identified in end-exercise heart rate at the global level, these effects were limited in magnitude and did not persist beyond the immediate post-exercise phase, reducing their physiological relevance. Taken together, these findings suggest that internal load remained relatively stable across conditions in line with contemporary models that conceptualise perceived exertion as an integrated signal modulated by physiological strain and contextual influences [10].

Perceived exertion provided one of the clearest applied signals. In males, perceived exertion was higher during individual trials than in group conditions, particularly when compared with the eight-person crew configuration. This finding indicates that task format influenced the subjective experience of effort. One possible interpretation is that individual and collective conditions differ in how effort is perceived, although the psychosocial mechanisms underlying this pattern were not directly elucidated [11,12].

Nevertheless, this perceptual difference was not accompanied by a consistent reduction in objective performance in larger crews. Therefore, the observed pattern does not provide strong evidence for a classical Ringelmann-type reduction in individual contribution [28] but rather indicates context-dependent variation in perceived effort without a corresponding and systematic reduction in objective output.

In females, a different profile was observed. The stability of perceived exertion across conditions, together with the emergence of indications of physiological–behavioural coupling in contexts of high interdependence, may be discussed in relation to Köhler-type interpretations, although the present evidence is indirect [13,14,29]. Nevertheless, given that objective performance changes between conditions were small, the most prudent interpretation is to avoid treating these findings as direct evidence of either Köhler or Ringelmann effects.

The correlational analyses add further nuance to this interpretation. The observed associations were weak, indicating that performance depends on multiple factors and that bivariate relationships account for a limited proportion of the variance. Even so, the presence of condition- and sex-specific couplings suggests that the relationship between pacing behaviour, physiological strain, and perceived effort may vary across task formats and between sexes. This reinforces an explanation based on effort regulation and self-regulation rather than on direct mechanical changes [10].

From a practical standpoint, the findings indicate that crew size primarily shapes the regulation and perception of effort, rather than leading to consistent changes in objective performance. In practice, collective configurations, particularly those involving larger crews, could be used strategically to modulate the subjective load of high-intensity work, especially in males, without necessarily compromising output. In contrast, for females, it may be useful to pay attention to pacing coordination and task structure in contexts involving greater collective demands [15,16,17].

The results support the conclusion that neither the Ringelmann effect nor the Köhler effect is expressed consistently as robust changes in objective performance under the experimental conditions employed. Instead, the most reproducible signals emerge in stroke rate and perceived exertion, suggesting context-dependent adjustments in execution and perceived effort in young rowers [11,14,27].

## 5. Conclusions

The present study suggests that sex-related differences in performance may be attenuated when technical level and training experience are comparable, particularly in contexts of structured cooperation. Despite the well-documented physiological differences [1,2,3], young male and female rowers exhibited broadly similar performance patterns across individual and group conditions. These findings suggest that, in collective endurance tasks, performance may be influenced not only by physiological factors but also by contextual and regulatory processes.

Group tests were characterised by an increase in stroke rate without proportional improvements in mechanical performance (distance and power), suggesting that adaptation to teamwork may be associated more with coordinative demands and effort regulation than with direct gains in objective performance. Although the observed effects were small in magnitude, this pattern is consistent with the trained and relatively homogeneous profile of the sample, in which behavioural variability is typically more limited.

Some differences were observed in perceived exertion across conditions and, in some cases, between sexes. In particular, males reported higher perceived exertion during individual trials than in group conditions. In contrast, females maintained relatively stable perceived exertion across conditions. However, these findings should be interpreted cautiously, as the present data do not allow firm conclusions regarding greater engagement or the psychosocial mechanisms underlying these patterns. Overall, the results support a cautious interpretation: condition-related effects appear to manifest in a context-dependent manner, influencing the perception and regulation of effort more clearly than objective performance outcomes.

From an applied perspective, these findings may help sport scientists, coaches, and athletes to make more informed use of individual and group conditions in youth rowing training programmes. Individual conditions may be more appropriate when the aim is to assess or train pacing, individual stroke regulation, and perceived exertion without the influence of crew dynamics, whereas group conditions may be useful for developing coordination, collective pacing, and adaptation to shared task demands.

### Limitations and Future Research Directions

Several limitations of the present study should be acknowledged when interpreting the results. First, the sample size was moderate, potentially limiting the generalisability of the findings. Second, the tests were performed using a rowing ergometer. Although this method provides a highly controlled experimental environment, transferability to on-water rowing is limited, as real boat conditions involve additional factors such as environmental conditions, stability, and the specific coordinative and social demands of collective work.

In addition, the design involving multiple conditions assessed across different sessions may have been influenced by familiarisation effects, variations in motivation, and potential accumulated fatigue. Moreover, the individual condition was administered first to balance performance levels and form comparable crews; therefore, potential order effects cannot be completely ruled out.

Furthermore, potentially relevant variables such as biological maturation, menstrual cycle, or body composition were not controlled, all of which may influence both performance and perceived exertion. In this regard, future research should prioritise the inclusion of direct psychological measures (e.g., task cohesion, perceived responsibility, indispensability, or motivation), together with physiological, hormonal, and emotional measures, in order to better understand the factors associated with sex-related differences and changes in effort regulation and stroke rate.

Importantly, the present study did not directly test Ringelmann or Köhler mechanisms, but rather examined performance outcomes and perceptual responses that may be interpreted in relation to these frameworks. Therefore, any interpretation based on these mechanisms should remain cautious and provisional.

With respect to future research, the inclusion of direct psychological measures (for example, perceived cohesion, motivation, responsibility/indispensability) should be a priority, as without them it is not possible to draw firm conclusions about the motivational or psychosocial processes underlying the observed changes in stroke rate and perceived exertion.

Considering sex and group dynamics in an integrated manner may help advance towards a more comprehensive framework for understanding collective performance during key stages of athletic development.

Finally, extending this approach to other cooperative sports (such as synchronised swimming, team canoeing, or tandem cycling) and to mixed-sex contexts would be pertinent, in order to examine whether the performance and perceptual patterns observed here are maintained or modified as a function of task type and degree of functional interdependence.

## Figures and Tables

**Table 1:** Descriptive statistics for the main study variables (mean ± SD).

Variable	Sex	C1	C2	C3	C4
Performance (strokes/min)	Men (*n* = 96)	33.40 ± 2.56	33.67 ± 2.36	34.36 ± 2.53	34.39 ± 2.18
	Women (*n* = 32)	32.47 ± 2.40	32.34 ± 1.68	31.94 ± 2.21	33.59 ± 2.21
	Total sample (*n* = 128)	33.16 ± 2.54	33.34 ± 2.28	33.76 ± 2.66	34.19 ± 2.21
Performance (m/min)	Men (*n* = 96)	863.88 ± 45.10	854.57 ± 52.83	865.07 ± 51.07	863.26 ± 47.63
	Women (*n* = 32)	768.25 ± 36.37	773.53 ± 39.36	769.75 ± 43.08	771.84 ± 36.90
	Total sample (*n* = 128)	839.97 ± 59.77	834.31 ± 60.88	841.24 ± 64.20	840.41 ± 60.07
Performance (W)	Men (*n* = 95)	311.705 ± 46.43	304.59 ± 53.10	314.74 ± 52.03	311.44 ± 50.43
	Women (*n* = 32)	219.109 ± 30.83	222.53 ± 36.64	220.87 ± 35.96	220.03 ± 30.59
	Total sample (*n* = 127)	288.374 ± 58.92	283.91 ± 60.93	291.09 ± 63.33	288.41 ± 60.95
End-exercise heart rate (bpm)	Men (*n* = 94)	184.98 ± 14.75	186.49 ± 11.95	186.40 ± 9.39	183.31 ± 11.25
	Women (*n* = 32)	186.25± 10.40	185.56 ± 10.25	184.00 ± 11.77	181.66 ± 12.87
	Total sample (*n* = 126)	185.30 ± 13.77	186.25 ± 11.51	185.79 ± 10.06	182.89 ± 11.65
Heart rate 3 min post-exercise (bpm)	Men (*n* = 94)	116.88 ± 10.14	116.59 ± 9.50	116.99 ± 11.93	115.03 ± 9.92
	Women (*n* = 32)	114.81 ± 14.29	118.50 ± 10.90	118.03 ± 9.20	117.84 ± 12.52
	Total sample (*n* = 126)	116.36 ± 11.31	117.07 ± 9.87	117.25 ± 11.27	115.75 ± 10.66
Perceived exertion (Borg scale)	Men (*n* = 95)	7.80 ± 0.79	7.63 ± 0.71	7.53 ± 0.73	7.46 ± 0.74
	Women (*n* = 32)	7.59 ± 0.84	7.47 ± 0.7	7.53 ± 0.8	7.56 ± 0.76
	Total sample (*n* = 127)	7.75 ± 0.81	7.59 ± 0.73	7.53 ± 0.74	7.49 ± 0.74

Mean scores and standard deviations for all outcome variables. bpm = beats per minute, SD = standard deviation.

**Table 2:** Within-subject effects from repeated-measures ANOVA with sphericity corrections.

	Sphericity Test			Huynh–Feldt Correction			
A. Within-subject factor: condition (C1, C2, C3, C4)	**Dependent variable**	**P Mauchly**	**GG ε**	**F**	***p***	**ɳ** **^2^**	**1 − β**
	Performance (strokes/min)	*p* < 0.001	0.912	F_(2,792)_ = 9.191	*p* < 0.001	0.035	0.995
	Performance (m/min)	*p* < 0.01	0.890	F_(2,722)_ = 6.536	*p* < 0.001	0.025	0.961
	Performance (W)	*p* < 0.001	0.815	F_(2,492)_ = 5.123	*p* = 0.003	0.02	0.881
	End-exercise heart rate (bpm)	*p* = 0.017	0.967	F_(2,962)_ = 4.610	*p* = 0.003	0.18	0.890
	Heart rate 3 min post-exercise (bpm)	*p* = 0.340	0.986	F_(3,000)_ = 1.432	*p* = 0.232	0.006	0.381
	Perceived exertion (Borg scale)	*p* = 0.006	0.958	F_(2,933)_ = 2.874	*p* = 0.037	0.11	0.681
B. Within-subject factor: condition (C1, C2, C3, C4) × between-subjects factor: sex (men, women)	**Dependent variable**	**P Mauchly**	**GG ε**	**F**	***p***	**ɳ** **^2^**	**1 − β**
	Performance (strokes/min)	*p* < 0.001	0.912	F_(5,583)_ = 1.506	*p* = 0.178	0.012	0.565
	Performance (m/min)	*p* < 0.001	0.890	F_(5,444)_ = 4.514	*p* < 0.001	0.034	0.980
	Performance (W)	*p* < 0.001	0.815	F_(4,983)_ = 2.140	*p* = 0.059	0.017	0.706
	End-exercise heart rate (bpm)	*p* = 0.017	0.967	F_(5,924)_ = 0.316	*p* = 0.927	0.003	0.139
	Heart rate 3 min post-exercise (bpm)	*p* = 0.340	0.986	F_(6,000)_ = 0.757	*p* = 0.604	0.006	0.303
	Perceived exertion (Borg scale)	*p* = 0.006	0.958	F_(5,866)_ = 0.439	*p* = 0.849	0.03	0.180

GG ε = Greenhouse–Geisser epsilon; bpm = beats per minute; ɳ^2^ = effect size; 1 − β = statistical power.

## Data Availability

The data presented in this study are not publicly available due to privacy restrictions.
